# Supplementary material for: Development and validation of the Japanese version of the Lesbian, Gay, Bisexual, and Transgender Development of Clinical Skills Scale
Source: PLoS One. 2024 Mar 27;19(3):e0298574. doi: 10.1371/journal.pone.0298574 (PMC10971768; doi:10.1371/journal.pone.0298574)
Supplement: S1 Appendix — (PDF) [file pone.0298574.s001.pdf]

日本語版 LGBT-DOCSS

注）

■ この尺度の項目は、レズビアン・ゲイ・バイセクシュアル・トランスジェンダー（LGBT)のクライエントや患者に関する臨床的準備、態度、基礎知識を調べるためのものです。各項目について、あなたの「同意する」「同意しない」の程度を、以下の尺度を用いて評価してください。

■ なお、この尺度の項目は、主に性的指向（LGB=レズビアン・ゲイ・バイセクシュアル）または性自認（トランスジェンダー）のどちらかについて尋ねています。
２つの質問は、レズビアン・ゲイ・バイセクシュアル・トランスジェンダー（LGBT)のクライエントや患者全体に言及する包括的なものです。

■ なお、このアンケート中での用語の定義は以下のとおりとします。

・「性的指向」とは、どのような性の人に恋愛的・性愛的に惹かれるかを意味します。
・「レズビアン」とは、女性を性的指向の対象とする女性を意味します。
・「ゲイ」とは、男性を性的指向の対象とする男性を意味します。
・「バイセクシュアル」とは、女性と男性の両方を性的指向の対象とする人を意味します。

・「性自認」とは、自分自身の性をどのように認識しているかを意味します。
・「トランスジェンダー」とは、出生時に割り当てられた性別とは異なる性自認をもつ人を意味します。
・「シスジェンダー」とは、出生時に割り当てられた性別と同じ性自認をもつ人を意味します。

・「LGBT」とは、レズビアン・ゲイ・バイセクシュアル・トランスジェンダーの頭文字をとった用語です。広義にはセクシュアルマイノリティの総称を意味することもありますが、ここではレズビアン・ゲイ・バイセクシュアル・トランスジェンダーの 4 つの性のあり方を意味することとします。

■　以下のそれぞれの項目について、当てはまる数字（１・２・３・４・５・６・７）のいずれか１つに◯をつけてください。

1.　トランスジェンダーの人々が医療サービスを利用しづらくする、規則や慣習などの制度的な障壁があることを私は知っている。

全く知らない			どちらともいえない			とてもよく知っている
1	2	3	4	5	6	7

2.　レズビアン・ゲイ・バイセクシュアルの人々が医療サービスを利用しづらくする、規則や慣習などの制度的な障壁があることを私は知っている。

全く知らない			どちらともいえない			とてもよく知っている
1	2	3	4	5	6	7

3.　トランスジェンダーであることは精神障害だと私は思う。

全くそう思わない			どちらともいえない			とてもそう思う
1	2	3	4	5	6	7

4.　LGBT のクライエントや患者と性的指向や性自認に関することを話すには、私は準備不足だと感じる。

全くそう思わない			どちらともいえない			とてもそう思う
1	2	3	4	5	6	7

5.　2 人の男性同士あるいは女性同士の同性間の関係は、男女間の関係ほど強くなく、確かでもない。

全くそう思わない			どちらともいえない			とてもそう思う
1	2	3	4	5	6	7

6.　レズビアン・ゲイ・バイセクシュアルの人々が、異性愛者の人々と比べて不当なほど多くの健康問題やメンタルヘルスの問題を経験しているという調査を私は知っている。

全く知らない			どちらともいえない			とてもよく知っている
1	2	3	4	5	6	7

7. レズビアン・ゲイ・バイセクシュアルの人は、子どもたちのいる前では自身の性的指向に関する言動を慎まなくてはならない。

全くそう思わない			どちらともいえない			とてもそう思う
1	2	3	4	5	6	7

8. トランスジェンダーの人々が、シスジェンダーの人々と比べて不当なほど多くの健康問題やメンタルヘルスの問題を経験しているという調査を私は知っている。

全く知らない			どちらともいえない			とてもよく知っている
1	2	3	4	5	6	7

9. トランスジェンダーの人々は、道徳的に逸脱していると私は思う。

全くそう思わない			どちらともいえない			とてもそう思う
1	2	3	4	5	6	7

10. 私は、トランスジェンダーのクライエントや患者を担当するための適切な臨床でのトレーニングや指導（スーパービジョン）を受けてきた。

全くそう思わない			どちらともいえない			とてもそう思う
1	2	3	4	5	6	7

11. 私は、レズビアン・ゲイ・バイセクシュアルのクライエントや患者を担当するための適切な臨床でのトレーニングや指導（スーパービジョン）を受けてきた。

全くそう思わない			どちらともいえない			とてもそう思う
1	2	3	4	5	6	7

12. レズビアン・ゲイ・バイセクシュアルの人のライフスタイルは不自然あるいは不道徳だ。

全くそう思わない			どちらともいえない			とてもそう思う
1	2	3	4	5	6	7

13. 私はレズビアン・ゲイ・バイセクシュアルのクライエントや患者を担当した経験がある。

全くない			どちらともいえない			とてもよくある
1	2	3	4	5	6	7

14. 臨床現場において、私はレズビアン・ゲイ・バイセクシュアルの人のアセスメントをする能力があると感じる。

全くそう思わない			どちらともいえない			とてもそう思う
1	2	3	4	5	6	7

15. 臨床現場において、私はトランスジェンダーの人のアセスメントをする能力があると感じる。

全くそう思わない			どちらともいえない			とてもそう思う
1	2	3	4	5	6	7

16. 私はトランスジェンダーのクライエントや患者を担当した経験がある。

全くない			どちらともいえない			とてもよくある
1	2	3	4	5	6	7

17. 生物学的な性別と反対の服装をする人たちは性的に異常だ。

全くそう思わない			どちらともいえない			とてもそう思う
1	2	3	4	5	6	7

18. LGBT のクライエントや患者を担当するのは、道徳的に不快だ。

全くそう思わない			どちらともいえない			とてもそう思う
1	2	3	4	5	6	7
