# Supplementary material for: Development and validation of the Japanese version of the Lesbian, Gay, Bisexual, and Transgender Development of Clinical Skills Scale
Source: PLoS One. 2024 Mar 27;19(3):e0298574. doi: 10.1371/journal.pone.0298574 (PMC10971768; doi:10.1371/journal.pone.0298574)
Supplement: S1 File — (DOCX) [file pone.0298574.s002.docx]

**LGBT-DOCSS**

Instructions: Items on this scale are intended to examine clinical preparedness, attitudes, and basic knowledge regarding lesbian, gay, bisexual, and transgender (LGBT) clients/patients. Please use the provided scale to rate your level of agreement or disagreement for each item. Please note, items on this scale primarily inquire about either sexual orientation (LGB = lesbian, gay, and bisexual) or gender identity (transgender). Two questions are inclusive and refer collectively to lesbian, gay, bisexual, and transgender (LGBT) clients/patients.

1. I am aware of institutional barriers that may inhibit transgender people from using health care services.

| Strongly  Disagree |  |  | Somewhat Agree/Disagree |  |  | Strongly Agree |
| --- | --- | --- | --- | --- | --- | --- |
| **1** | 2 | 3 | **4** | 5 | 6 | **7** |

2. I am aware of institutional barriers that may inhibit LGB people from using health services.

| Strongly  Disagree |  |  | Somewhat Agree/Disagree |  |  | Strongly Agree |
| --- | --- | --- | --- | --- | --- | --- |
| **1** | 2 | 3 | **4** | 5 | 6 | **7** |

3. I think being transgender is a mental disorder.

| Strongly  Disagree |  |  | Somewhat Agree/Disagree |  |  | Strongly Agree |
| --- | --- | --- | --- | --- | --- | --- |
| **1** | 2 | 3 | **4** | 5 | 6 | **7** |

4. I would feel unprepared talking with a LGBT client/patient about issues related to their sexual orientation or gender identity.

| Strongly  Disagree |  |  | Somewhat Agree/Disagree |  |  | Strongly Agree |
| --- | --- | --- | --- | --- | --- | --- |
| **1** | 2 | 3 | **4** | 5 | 6 | **7** |

5. A same sex relationship between two men or two women is not as strong and as committed as one between a man and a woman.

| Strongly  Disagree |  |  | Somewhat Agree/Disagree |  |  | Strongly Agree |
| --- | --- | --- | --- | --- | --- | --- |
| **1** | 2 | 3 | **4** | 5 | 6 | **7** |

6. I am aware of research indicating that LGB individuals experience disproportionate levels of health and mental health problems compared to heterosexual individuals.

| Strongly  Disagree |  |  | Somewhat Agree/Disagree |  |  | Strongly Agree |
| --- | --- | --- | --- | --- | --- | --- |
| **1** | 2 | 3 | **4** | 5 | 6 | **7** |

7. LGB individuals must be discreet about their sexual orientation around children.

| Strongly  Disagree |  |  | Somewhat Agree/Disagree |  |  | Strongly Agree |
| --- | --- | --- | --- | --- | --- | --- |
| **1** | 2 | 3 | **4** | 5 | 6 | **7** |

8. I am aware of research indicating that transgender individuals experience disproportionate levels of health and mental health problems compared to cisgender individuals.

| Strongly  Disagree |  |  | Somewhat Agree/Disagree |  |  | Strongly Agree |
| --- | --- | --- | --- | --- | --- | --- |
| **1** | 2 | 3 | **4** | 5 | 6 | **7** |

9. When it comes to transgender individuals, I believe they are morally deviant.

| Strongly  Disagree |  |  | Somewhat Agree/Disagree |  |  | Strongly Agree |
| --- | --- | --- | --- | --- | --- | --- |
| **1** | 2 | 3 | **4** | 5 | 6 | **7** |

10. I have received adequate clinical training and supervision to work with transgender clients/patients.

| Strongly  Disagree |  |  | Somewhat Agree/Disagree |  |  | Strongly Agree |
| --- | --- | --- | --- | --- | --- | --- |
| **1** | 2 | 3 | **4** | 5 | 6 | **7** |

11. I have received adequate clinical training and supervision to work with lesbian, gay, and bisexual (LGB) clients/patients

| Strongly  Disagree |  |  | Somewhat Agree/Disagree |  |  | Strongly Agree |
| --- | --- | --- | --- | --- | --- | --- |
| **1** | 2 | 3 | **4** | 5 | 6 | **7** |

12. The lifestyle of a LGB individual is unnatural or immoral.

| Strongly  Disagree |  |  | Somewhat Agree/Disagree |  |  | Strongly Agree |
| --- | --- | --- | --- | --- | --- | --- |
| **1** | 2 | 3 | **4** | 5 | 6 | **7** |

13. I have experience working with LGB clients/patients.

| Strongly  Disagree |  |  | Somewhat Agree/Disagree |  |  | Strongly Agree |
| --- | --- | --- | --- | --- | --- | --- |
| **1** | 2 | 3 | **4** | 5 | 6 | **7** |

14. I feel competent to assess a person who is LGB in a therapeutic setting.

| Strongly  Disagree |  |  | Somewhat Agree/Disagree |  |  | Strongly Agree |
| --- | --- | --- | --- | --- | --- | --- |
| **1** | 2 | 3 | **4** | 5 | 6 | **7** |

15. I feel competent to assess a person who is transgender in a therapeutic setting.

| Strongly  Disagree |  |  | Somewhat Agree/Disagree |  |  | Strongly Agree |
| --- | --- | --- | --- | --- | --- | --- |
| **1** | 2 | 3 | **4** | 5 | 6 | **7** |

16. I have experience working with transgender clients/patients.

| Strongly  Disagree |  |  | Somewhat Agree/Disagree |  |  | Strongly Agree |
| --- | --- | --- | --- | --- | --- | --- |
| **1** | 2 | 3 | **4** | 5 | 6 | **7** |

17. People who dress opposite to their biological sex have a perversion.

| Strongly  Disagree |  |  | Somewhat Agree/Disagree |  |  | Strongly Agree |
| --- | --- | --- | --- | --- | --- | --- |
| **1** | 2 | 3 | **4** | 5 | 6 | **7** |

18. I would be morally uncomfortable working with a LGBT client/patient.

| Strongly  Disagree |  |  | Somewhat Agree/Disagree |  |  | Strongly Agree |
| --- | --- | --- | --- | --- | --- | --- |
| **1** | 2 | 3 | **4** | 5 | 6 | **7** |
