# Supplementary figures and images for: Development and validation of the Japanese version of the Lesbian, Gay, Bisexual, and Transgender Development of Clinical Skills Scale
Source: PLoS One. 2024 Mar 27;19(3):e0298574. doi: 10.1371/journal.pone.0298574 (PMC10971768; doi:10.1371/journal.pone.0298574)

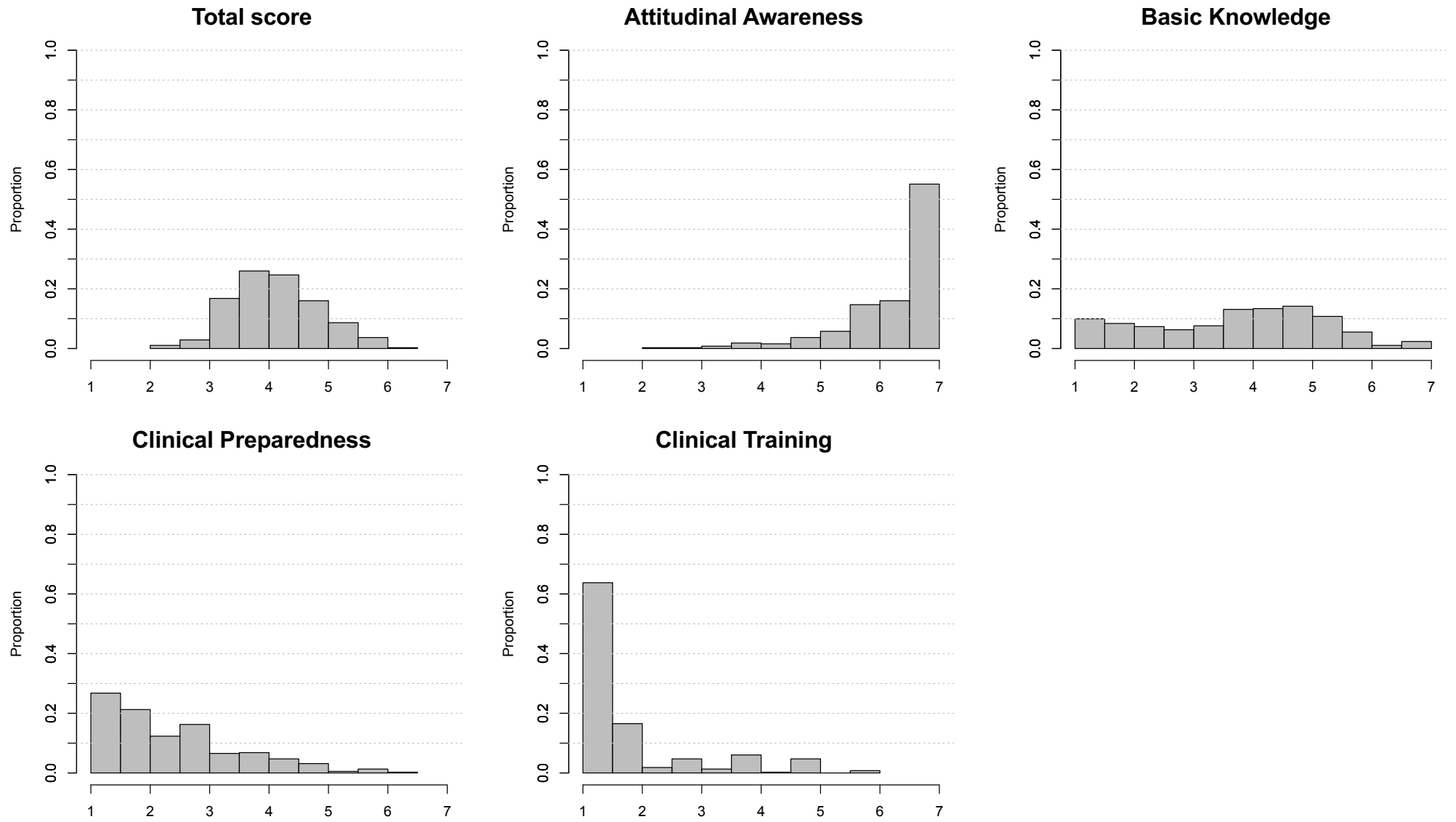

**S1 Fig. The score distributions for the overall and each subscale**

Supplement: S1 Fig — (PDF) [file pone.0298574.s003.pdf]
