# Supplementary material for: Development and validation of the Japanese version of the Lesbian, Gay, Bisexual, and Transgender Development of Clinical Skills Scale
Source: PLoS One. 2024 Mar 27;19(3):e0298574. doi: 10.1371/journal.pone.0298574 (PMC10971768; doi:10.1371/journal.pone.0298574)
Supplement: S1 Table — (PDF) [file pone.0298574.s004.pdf]

S1 Table. The scores of cisgender participants and each professional healthcare specialization

	No.	Total (mean (SD))	Attitudinal Awareness (mean (SD))	Basic Knowledge (mean (SD))	Clinical Preparedness (mean (SD))	Clinical Training (mean (SD))
Cisgender participants (n = 371)						
Cisgender male	106	4.02 (0.81)	6.02 (0.99)	3.57 (1.56)	2.43 (1.16)	1.89 (1.41)
Cisgender female	265	4.17 (0.68)	6.40 (0.73)	3.88 (1.44)	2.30 (1.11)	1.67 (1.09)
Professional healthcare specialization (n = 381)						
Physician (including dentist)	89	4.40 (0.88)	6.34 (0.87)	4.13 (1.71)	2.80 (1.31)	2.18 (1.62)
Nurse	252	4.13 (0.68)	6.31 (0.80)	3.84 (1.43)	2.29 (1.07)	1.65 (1.02)
Pharmacist	34	3.66 (0.44)	6.06 (0.88)	3.03 (1.28)	1.75 (0.68)	1.26 (0.85)
Licensed or certified clinical psychologist	6	4.71 (0.49)	6.86 (0.22)	4.42 (1.10)	2.90 (1.43)	2.33 (1.21)

Note. LGBT-DOCSS-JP total scale scores and subscale scores are described. SD, standard deviation.
