# Supplementary material for: Development and validation of the Japanese version of the Lesbian, Gay, Bisexual, and Transgender Development of Clinical Skills Scale
Source: PLoS One. 2024 Mar 27;19(3):e0298574. doi: 10.1371/journal.pone.0298574 (PMC10971768; doi:10.1371/journal.pone.0298574)
Supplement: S2 Table — (PDF) [file pone.0298574.s005.pdf]

S2 Table. Hypothesis testing among cisgender-heterosexual participants (n = 293)

	No.	Total (mean (SD))	Attitudinal Awareness (mean (SD))	Basic Knowledge (mean (SD))	Clinical Preparedness (mean (SD))	Clinical Training (mean (SD))
Homosexual friends/family						
Yes	41	4.31 (0.81)	6.45 (0.97)	3.78 (1.56)	2.71 (1.38)	1.93 (1.45)
Probably	14	4.33 (0.57)	6.44 (0.42)	4.71 (1.29)	2.19 (1.02)	1.57 (1.00)
Probably not	135	4.10 (0.72)	6.28 (0.81)	3.66 (1.51)	2.33 (1.15)	1.81 (1.30)
No	103	3.95 (0.62)	6.15 (0.89)	3.59 (1.28)	2.11 (0.99)	1.55 (1.04)
P value		**< 0.01**	**< 0.01**	0.09	**0.04**	0.11
Homosexual coworker						
Yes	74	4.45 (0.78)	6.43 (0.8)	4.07 (1.57)	2.89 (1.31)	2.23 (1.60)
Probably	72	4.11 (0.69)	6.28 (0.91)	3.73 (1.39)	2.38 (1.10)	1.58 (1.08)
Probably not	120	3.87 (0.61)	6.13 (0.87)	3.48 (1.40)	1.96 (0.89)	1.53 (0.99)
No	27	4.00 (0.48)	6.36 (0.71)	3.62 (1.28)	1.96 (1.06)	1.59 (1.01)
P value		**< 0.01**	**0.02**	**< 0.01**	**< 0.01**	**< 0.01**
Trans friends/family						
Yes	17	4.74 (0.89)	6.46 (0.87)	4.65 (1.27)	3.08 (1.51)	3.00 (1.87)
Probably	11	4.44 (0.76)	6.57 (0.36)	4.32 (1.79)	2.53 (1.24)	2.00 (1.41)
Probably not	149	4.10 (0.69)	6.33 (0.78)	3.60 (1.51)	2.34 (1.17)	1.67 (1.15)
No	116	3.95 (0.63)	6.11 (0.95)	3.65 (1.30)	2.10 (0.96)	1.57 (1.08)
P value		**< 0.01**	**0.01**	0.10	**0.02**	**< 0.01**
Trans coworker						
Yes	32	4.37 (0.73)	6.18 (1.07)	4.05 (1.22)	3.06 (1.01)	1.95 (1.40)
Probably	23	4.30 (0.86)	6.34 (0.87)	4.12 (1.56)	2.57 (1.45)	1.89 (1.45)
Probably not	171	4.02 (0.72)	6.23 (0.88)	3.57 (1.51)	2.21 (1.11)	1.74 (1.23)
No	67	4.05 (0.54)	6.36 (0.66)	3.74 (1.30)	2.07 (0.99)	1.52 (1.02)
P value		0.06	0.99	0.22	**< 0.01**	0.10

Note. LGBT-DOCSS-JP total scale scores and subscale scores are described. All statistical tests used the Jonckheere–Terpstra test. Bold font shows significance at $P < 0.05$ in P value line. SD, standard deviation.
